# Spectrochemical analysis of liquid biopsy harnessed to multivariate analysis towards breast cancer screening

**DOI:** 10.1038/s41598-020-69800-7

**Published:** 2020-07-30

**Authors:** Daniel L. D. Freitas, Ingrid M. Câmara, Priscila P. Silva, Nathália R. S. Wanderley, Maria B. C. Alves, Camilo L. M. Morais, Francis L. Martin, Tirzah B. P. Lajus, Kassio M. G. Lima

**Affiliations:** 1 Institute of Chemistry, Biological Chemistry and Chemometrics, Federal University of Rio Grande do Norte, Natal, 59072-970 Brazil; 2 Departamento de Biologia Celular e Genética – Serviço de Aconselhamento Genético, Centro de Oncologia Avançado/CECAN, Universidade Federal do Rio Grande do Norte – Hospital Liga Contra o Câncer, Natal, Brazil; 3 Departament of Genetics and Cell Biology, Centro de Biociências, Federal University of Rio Grande do Norte, Natal, 59072-970 Brazil; 4 Departament of Pharmacy, Centro de Ciências da Saúde, Federal University of Rio Grande do Norte, Natal, 59072-970 Brazil; 5 Lancashire Teaching Hospitals NHS Trust, Royal Preston Hospital, Fulwood, Preston, PR2 9HT UK; 6 School of Pharmacy and Biomedical Sciences, University of Central Lancashire, Preston, PR1 2HE UK

**Keywords:** Infrared spectroscopy, Breast cancer

## Abstract

Mortality due to breast cancer could be reduced via screening programs where preliminary clinical tests employed in an asymptomatic well-population with the objective of identifying cancer biomarkers could allow earlier referral of women with altered results for deeper clinical analysis and treatment. The introduction of well-population screening using new and less-invasive technologies as a strategy for earlier detection of breast cancer is thus highly desirable. Herein, spectrochemical analyses harnessed to multivariate classification techniques are used as a bio-analytical tool for a Breast Cancer Screening Program using liquid biopsy in the form of blood plasma samples collected from 476 patients recruited over a 2-year period. This methodology is based on acquiring and analysing the spectrochemical fingerprint of plasma samples by attenuated total reflection Fourier-transform infrared spectroscopy; derived spectra reflect intrinsic biochemical composition, generating information on nucleic acids, carbohydrates, lipids and proteins. Excellent results in terms of sensitivity (94%) and specificity (91%) were obtained using this method in comparison with traditional mammography (88–93% and 85–94%, respectively). Additional advantages such as better disease prognosis thus allowing a more effective treatment, lower associated morbidity, fewer false-positive and false-negative results, lower-cost, and higher analytical frequency make this method attractive for translation to the clinical setting.

## Introduction

Breast cancer is the second most common and the leading cause of cancer-related death amongst women^1^. According to the Brazilian Mortality Information System, 14,206 women died in 2013 due to this disease^2^. In 2014, the estimation was about 49,240 cases, and in 2018 it was expected to reach 59,700 new cases of breast cancer in Brazil alone^1^. This neoplasm is relatively rare in women < 35 years old, and increases progressively above this age, especially after age 50 years^3^. Therefore, breast cancer is a major public health problem taking into consideration the detection and treatment costs^4^. The control of breast cancer has been a priority and is present in the Brazilian Strategic Action Plan for Confronting Non-transmissible Chronic Diseases since 2011^5^.

Only one in three cases of breast cancer can be cured if discovered at an early stage^2^ and there are no effective ways of reducing the incidence of this disease^6^. The best alternative approach to tackle breast cancer is the concept that the earlier the disease is detected, the more effective is the treatment. Early detection through screening is the only method that has proven to be effective in reducing mortality^1^. Screening programs are an important health policy practice where the asymptomatic phase of disease is long enough to allow direct or indirect detection of pre-cancerous lesions. A significant degree of transformation in such lesions found in this phase would allow determination of their clinical significance and implementation of effective treatment to improve the patient’s prognosis. Such a screening test that diagnoses early disease needs to be acceptable to patients and available at a reasonable cost^5^.

Mammography is the recommended method for routine screening of breast cancer worldwide^6^. This technique performed with an x-ray machine is described as a radiological examination for evaluation of the breasts. It can be used for checking breast cancer-like lesions in apparently healthy woman by finding nodules or calcifications. Exposure to this radiation rarely causes cancer, unless performed with a high periodic frequency whereby risk will increase. Besides being considered painful, relatively expensive, and a source of much discomfort and even embarrassment to patients, its sensitivity varies from 88 to 93%, while its specificity varies from 85 to 94%^6^. Such statistical metrics demonstrate the proportion of women with breast cancer who will present a positive mammogram signalling disease presence, and the rate of women without breast cancer who will have a normal mammography, respectively^6^. Some breast cancer screening tests also include breast self-examination (BSE), clinical examination of breasts (CBE), nuclear magnetic resonance (NMR), and ultrasonography. However, the time from initial patient examination until diagnosis can be too lengthy; about 70% of breast cancer cases lead to complete removal of the breast(s). Many examinations are required to identify the presence of neoplasm: mammogram, breast exam, biopsy, magnetic resonance imaging (MRI) and ultrasound.

Infrared (IR) spectroscopy is a vibrational technique capable of analysing biomolecules, such as nucleic acids (asymmetric PO_2_^−^ in DNA and RNA at ~ 1,225 cm^−1^), carbohydrates (C–O stretching at ~ 1,155 cm^−1^), proteins (amide II at ~ 1,550 cm^−1^ and amide I at ~ 1,660 cm^−1^) and lipids (C=C stretching at ~ 1,750 cm^−1^), that exhibit characteristic features in the IR region^7^. Attenuated total reflection Fourier-transform IR (ATR-FTIR) spectroscopy has been used to analyse several biofluids due to its fast spectral acquisition, minimum sample preparation and sample volume, and its non-destructive nature to the sample^8^. Recent research is progressing gradually in which excellent diagnostic results compared to traditional methods have been obtained in various types of cancer such as ovarian^9^, cervical^10^, and prostate^11^; additionally, to diagnosis neurodegenerative diseases such as Alzheimer’s^12^. Herein, we present the results of using ATR-FTIR spectroscopy together with chemometrics for classification of patients with breast cancer in a large-scale screening program using blood biopsies.

## Results

**Figure 1:**
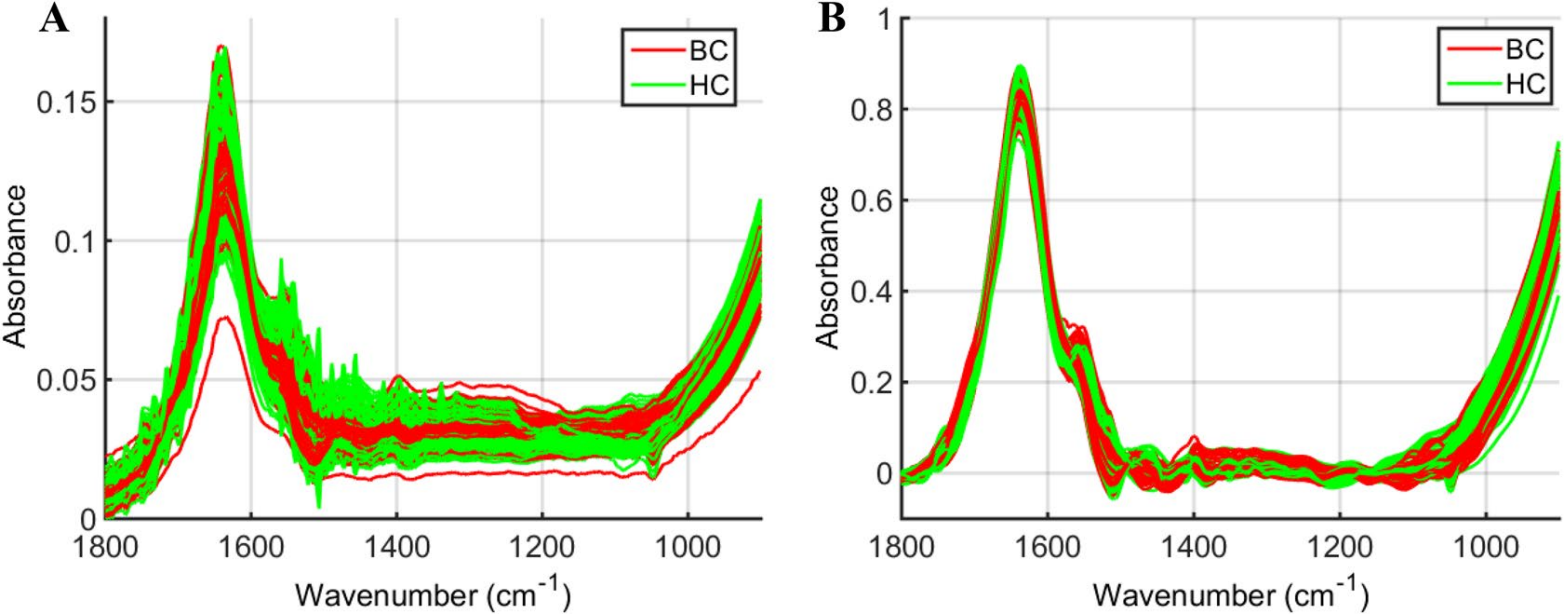
ATR-FTIR spectra of plasma samples in the bio-fingerprint region (1,800–900 cm^−1^). (**a**) Raw spectral data for breast cancer (BC) and healthy controls (HC) samples; (**b**) pre-processed spectral data (Savitzky–Golay smoothing [window of 7 points, 2nd order polynomial fitting] followed by AWLS baseline correction and normalization to the Amide I peak) for breast cancer (BC) and healthy controls (HC) samples.

**Figure 2:**
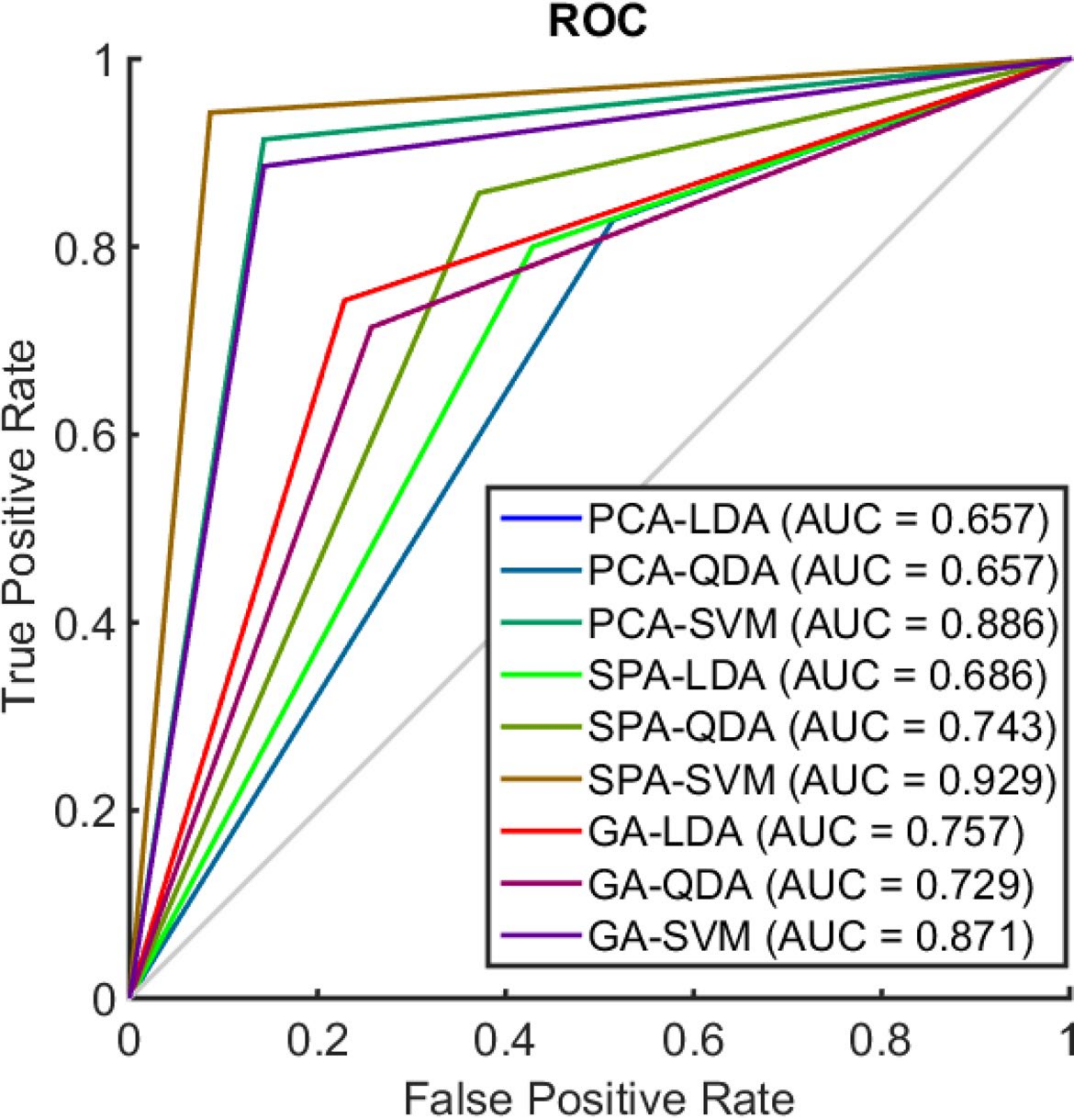
Receiver operating characteristic (ROC) curve. Where, PCA-LDA: principal component analysis linear discriminant analysis; PCA-QDA: principal component analysis quadratic discriminant analysis; PCA-SVM: principal component analysis support vector machines; SPA-LDA: successive projections algorithm linear discriminant analysis; SPA-QDA: successive projections algorithm quadratic discriminant analysis; SPA-SVM: successive projections algorithm support vector machines; GA-LDA: genetic algorithm linear discriminant analysis; GA-QDA: genetic algorithm quadratic discriminant analysis; GA-SVM: genetic algorithm support vector machines. AUC: area under the curve.

The FTIR spectral data in the fingerprint region (900–1,800 cm^−1^) were pre-processed by Savitzky–Golay smoothing (window of 7 points, 2nd order polynomial fitting) followed by AWLS baseline correction and normalization to the Amide I peak (1,650 cm^−1^). The raw and pre-processed spectral data are shown in Fig. 1, where visual overlaps between breast cancer and healthy control spectra are present throughout the whole spectral region indicating the need of chemometric techniques to distinguish samples in such complex matrices. The pre-processed spectral data underwent chemometric analysis by several classification techniques (Table 1). Amongst the classification techniques tested, SPA-SVM presented the best classification performance with accuracy of 92.9% (94% sensitivity and 91% specificity) to detect breast cancer samples based on an external test set (15% of samples, *n* = 71 patients). ~ 70% of samples (*n* = 334 patients) were used for model construction and another 15% for internal validation (*n* = 71 patients). Overall classification performance represented by the F-Score and G-Score values was good (93%), indicating equal performance with or without considering imbalanced data. Figure 2 shows the receiver operating characteristic (ROC) curve for all models. The best ROC curve (area under the curve [AUC] = 0.929) was found for SPA-SVM, indicating an excellent predictive performance. PCA-SVM (AUC = 0.886) and GA-SVM (AUC = 0.871) were, respectively, the second and third best classification algorithms, demonstrating a good classification performance.

**Table 1:** Statistical results in % for the test set using the PCA-LDA/QDA/SVM, SPA-LDA/QDA/SVM and GA-LDA/QDA/SVM to discriminate healthy controls and breast cancer samples.

Model	AC	SENS	SPEC	YOU	PPV	NPV	F-score	G-score
PCA-LDA	65.7	82.9	486	31.4	61.7	73.9	61.2	63.4
PCA-QDA	65.7	82.9	48.6	31.4	61.7	73.9	61.2	63.4
PCA-SVM	88.6	91.4	85.7	77.1	86.5	90.9	88.5	88.5
SPA-LDA	68.6	80.0	57.1	37.1	65.1	74.1	66.7	67.6
SPA-QDA	74.3	85.7	62.9	48.6	69.8	81.5	72.5	73.4
**SPA-SVM**	**92.9**	**94.3**	**91.4**	**85.7**	**91.7**	**94.1**	**92.8**	**92.8**
GA-LDA	75.7	74.3	77.1	51.4	76.5	75.0	75.7	75.7
GA-QDA	72.9	71.4	74.3	45.7	73.5	72.2	72.8	72.8
GA-SVM	87.1	88.6	85.7	74.3	86.1	88.2	87.1	87.1

AC, Accuracy; SENS, Sensitivity; SPEC, Specificity; YOU, Youden’s Index; PPV, Positive predictive value; NPV, Negative predictive value. The best model (SPA-SVM) is in bold.

**Figure 3:**
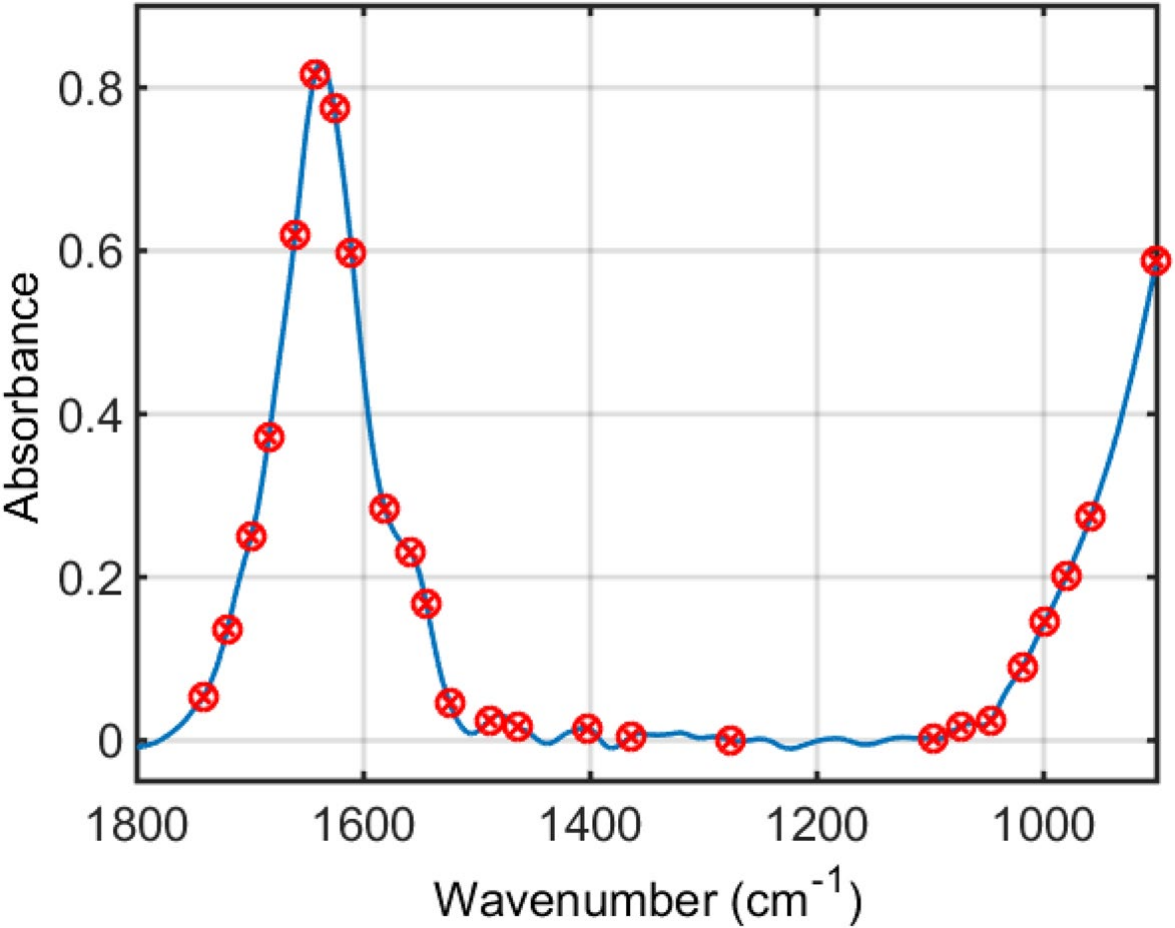
Selected wavenumbers by the successive projections algorithm support vector machines (SPA-SVM) model.

The spectral variables selected by the best classification model (SPA-SVM) are shown in Fig. 3. In total, 16 wavenumbers (901, 959, 980, 999, 1,018, 1,277, 1,364, 1,402, 1,464, 1,489, 1582, 1,311, 1626, 1643, 1661, and 1742 cm^−1^) were responsible for class differentiation using SPA-SVM. The tentative biochemical assignments of these variables based on Movasaghi et al*.*^13^ are shown in Table 2.

**Table 2:** Selected wavenumbers by the SPA-SVM to distinguish healthy controls and breast cancer samples.

Selected wavenumber (cm^−1^)	Tentative assignment
901	Phosphodiester (absorbances due to collagen and glycogen)
959	Symmetric stretching vibration of n_1_PO_4_
980	OCH_3_ (polysaccharides)
999	Ring stretching vibrations mixed strongly with CH in plane bending
1,018	n(CO), n(CC), d(OCH), ring (polysaccharides, pectin)
1,277	Vibrational modes of collagen
1,311	Amide III band components of proteins
1,364	Stretching C–O, deformation C–H, deformation N–H
1,402	Symmetric CH_3_ bending modes of the methyl groups of proteins
1,464	CH_2_ scissoring mode of the acyl chain of lipid
1,489	In-plane CH bending vibration
1582	Ring C–C stretch of phenyl
1626	Peak of nucleic acids due to the base carbonyl stretching and ring breathing mode
1643	Amide I band (arises from C=O stretching vibrations)
1661	n(C=C) cis in lipids and fatty acids
1742	C=O stretching mode of lipids

## Discussion

Breast cancer accounts for approximately 15% of all female cancer deaths and has a 5-years survival rate ranging from approximately 40% in low-income countries to ≥ 80% in developing contruies^14^. Its incidence is continually increasing worldwide. This is partly due to a change in the distribution of risk factors: e.g., in developed countries such as the UK, there have been significant increases in women giving birth later in life and in the number of women childless by age 45 years. In addition, there has been an increasing adoption of Westernized lifestyles in developing countries^14^, which may be a risk factor for breast cancer.

Mammography-based breast cancer screening is a common practice for early detection of breast cancers, where its efficiency has been demonstrated in randomized controlled trials and observational studies; hence, most organizations that issue recommendations endorse regular mammography as an important part of preventive care^15^. However, although mammography-based breast cancer screening is associated with reduced morbidity and mortality, the majority of women who undergo screening will not develop breast cancer in their lifetime^15^. In addition to the low risk of cumulative exposure to radiation over time and the great discomfort or shame associated with mammography-based screening, false positive results may lead to additional tests and investigations potentially causing psychological distress and anxiety. Conversely, negative results (i.e., where no signs of abnormality are found in the screening) may falsely reassure women when cancer is actually present^14^. Moreover, mammography-based screening may also not benefit all women who are diagnosed with breast cancer, since it may lead to harm in women who undergo further biopsy for abnormalities that may not be breast cancer^15^. For these reasons, less invasive and more accurate breast cancer screening strategies are urgently needed.

Herein, ATR-FTIR spectroscopy in conjunction with chemometric techniques was used to detect breast cancer in a total cohort of 476 patients recruited over 2 years for an early-stage breast cancer screening program in Natal, Brazil. Breast cancer detection among normal samples was successfully performed based on the blood plasma spectra with 93% accuracy (94% sensitivity, 91% specificity, AUC = 0.929) in an external (blind) cohort of 71 patients using the SPA-SVM algorithm. Sixteen spectral features were responsible for class differentiation in the fingerprint region (Table 2). These are predominantly associated with phosphodiesters (P–O vibrations), polysaccharides (C–O stretching), proteins (CH_3_ bending, Amide III, Amide I band), nucleic acids (C=O stretching and C–C ring breathing mode), and lipids (C=O stretching and (C=C)_cis_). C–O vibrations in carbohydrates, P–O vibrations in phosphodiesters, and proteins vibrations; these have been previously associated with breast cancer in serum^15,16^. Serum applications for breast cancer detection have been performed using IR spectroscopy by Backhaus et al*.*^15^, where 98% sensitivity and 95% specificity (using cluster analysis) and 92% sensitivity and 100% specificity [using artificial neural networks (ANN)] was obtained in a study carried out with 196 patients. Likewise, Elmi et al*.*^16^ detected breast cancer in serum-based IR spectroscopy with 76% sensitivity and 72% specificity for breast cancer cases using principal component analysis linear discriminant analysis (PCA-LDA) in a study with 86 samples (43 breast cancer, 43 healthy controls). The results reported herein are higher taking into consideration the large number of patients, where the sensitivity and specificity are found to be > 90%; being comparable to results obtained by more sophisticated methods such as using quantum cascade laser IR imaging, where sensitivity and specificity has been reported at 94% and 86%, respectively, using a random forest classifier^17^. However, there are no studies reporting breast cancer screening based on plasma samples using IR spectroscopy for a big cohort of samples. Herein, 476 patients were studied resulting in a diagnostic accuracy, sensitivity and specificity above 90% for cancer detection.

## Methods

### Samples

In this study, we evaluated two groups of women. The first, Breast Cancer (BC), refers to a group of women diagnosed with breast cancer, with or without neoadjuvant treatment, and were collected by professionals trained at the Liga Contra o Câncer Hospital (Natal/RN, Brazil), during a period of 2 years. The second, Healthy Controls (HC), refers to a group of women with no previous or current diagnosis of breast cancer, collected at the Prontoclínica Dr. Paulo Gurgel (Natal/RN, Brazil), during the same time period. In both groups, patients were > 18 years old, and family history related to some type of cancer was not taken into account. The Institutional Ethics Committee for Human Research of the Hospital Universitário Onofre Lopes (HUOL), of the Federal University of Rio Grande do Norte (UFRN), Brazil, approved this study (Ethical Approval Number—44113115.1.1001.5292) and informed consent was obtained from all subjects. Also, all the methods carried out in this study were by the approved guidelines. Samples from both groups were obtained after the reading of a Free Informed Consent Form and signature of the patients. Vacutainer tubes BD with 5 mL EDTA were used with disposable vacuum syringes. Thereafter, they were centrifuged for 10 min, and frozen at approximately − 20 °C until the time of analysis. A total of 476 samples were obtained.

### ATR-FTIR spectroscopy

The samples were removed from the freezer 15 min before analysis to allow thawing. Samples were randomized and, to minimize temporal or instrumental effects, a similar number of samples from both groups were measured on each day. The absorption spectra were obtained using an attenuated total reflection Fourier-transform infrared (ATR-FTIR) spectrometer model IRAffinity-1S (Shimadzu Corp., Kyoto, Japan). The spectra were obtained in the range between 600 and 4,000 cm^−1^, with 32 co-added scans and 4 cm^−1^ spectral resolution (2 cm^−1^ data spacing). The ATR crystal was cleaned with alcohol (70% v/v) and acetone (P.A.) for each new sample and before setting the new background. A 10-μL staken performed. This procedure was repeated in triplicate. The measurement time for each sample was approximately 5 min.

Three spectra collected per sample were first averaged and the following pre-processing was applied to the dataset: truncation to the biofingerprint region (900–1800 cm^−1^ with 468 wavenumber data points), Savitzky–Golay (SG) smoothing to remove random noise (window = 15 points, 2nd order polynomial fitting), automatic weighted least squares baseline correction, and normalization to the Amide I peak (1,650 cm^−1^).

### Data analysis

The spectral data import, pre-processing and construction of multivariate classification models were performed using the MATLAB R2014b environment version 8.4 (MathWorks, Inc., Natick, USA) with the PLS-Toolbox version 7.9.3 (Eigenvector Research, Inc., Manson, USA) and laboratory-made routines. All spectra were organized into a data matrix, where samples were represented as rows and the wavenumbers as columns. The samples were divided into three different subsets by the Kennard–Stone (KS) sample selection algorithm^18^: training (70%), validation (15%) and test (15%) sets. The training set was used to build the classification models, while the validation set to optimize and evaluate its internal performance. Finally, the test set was used to evaluate the model classification performance towards external samples.

The computational analysis consisted of testing three algorithms for feature extraction and selection: principal component analysis (PCA)^19^, successive projections algorithm (SPA)^20^ or genetic algorithm (GA)^21^; followed by discriminant analysis classifiers: linear discriminant analysis (LDA)^22^, quadratic discriminant analysis (QDA)^22^ or support vector machines (SVM)^23^. These algorithms were coupled as feature extraction/selection and classification as: PCA-LDA, PCA-QDA, and PCA-SVM; SPA-LDA, SPA-QDA, and SPA-SVM; and GA-LDA, GA-QDA, and GA-SVM.

PCA is a feature extraction method widely used for data reduction^19^. It decomposes the pre-processed spectral data into a small number of principal components (PCs) containing scores (variance on sample direction) and loadings (variance on wavenumber direction). The PCA scores are used to assess similarities/dissimilarities between the samples, while the PCA loadings to investigate potential spectral markers. SPA is a forward feature selection method^20^. Its purpose is to select wavenumbers whose information content is minimally redundant in order to solve co-linearity problems. The model starts with one wavenumber, then incorporates a new one at each iteration until it reaches a specified number of wavenumbers. SPA does not modify the original data space as PCA does. In SPA, the projections are used only for variable selection purposes. Thus, the relationship between the spectral variables is preserved.

On the other hand, the GA uses a combination of selection, recombination and mutation to select a set of variables^21^. The GA aims to reduce the original data in a few number of wavenumbers following a natural evolutionary process based on Darwin’s theory where the best set of wavenumbers, in this case considered as a chromosome, is selected according to a fitness function. The GA routine was carried out during 100 generations with 200 chromosomes each where mutation and crossover probabilities were set to 10% and 60%, respectively. The best solution in GA, in terms of fitness value, is obtained after three realizations starting from different random initial populations. Similarly to SPA, GA also does not modify the original data space as PCA does. The SPA/GA fitness is calculated as the inverse of the cost function $G$, which is defined as follows^24^:

1 $$ G = \frac{1}{{N_{V} }} \mathop \sum \limits_{n = 1}^{{N_{V} }} g_{n} $$

where $N_{V}$ is the number of validation samples and $g_{n}$ is defined as:

2 $$ g_{n} = \frac{{r^{2} \left( {x_{n} ,m_{I\left( n \right)} } \right)}}{{min_{I\left( m \right) \ne I\left( n \right)} r^{2} \left( {X_{n} ,m_{I\left( m \right)} } \right)}} $$

where the numerator is the squared Mahalanobis distance between object $x_{n}$ of class index $I\left( n \right)$ and the sample mean $m_{I\left( n \right)}$ of its true class; and the denominator is the squared Mahalanobis distance between object $x_{n}$ and the centre of the closest wrong class. The advantages of these variable reduction methods (PCA, SPA and GA) prior discriminant analysis lie in the fact that they efficiently remove co-linearity in the dataset, thus preserving only non-redundant information; they solve dimensionality problems for LDA and QDA; and they speed-up the computational time for SVM.

LDA and QDA are discriminant analysis classifiers based on a Mahalanobis distance calculation between the samples; where the main difference between them is that LDA assumes classes having similar variance structures, hence, using a pooled covariance matrix, while QDA assumes classes having different variance structures therefor using the variance–covariance matrix of each class individually for calculation^22^. The LDA classification score for sample *i* of class $k$ ($L_{ik}$) is calculated for a given class sample in a non-Bayesian form by the following equation^22,25^:

3 $$ L_{ik} = \left( {{\mathbf{x}}_{i} - { }{\overline{\mathbf{x}}}_{k} } \right)^{{\text{T}}} {\mathbf{C}}_{{{\text{pooled}}}}^{ - 1} \left( {{\mathbf{x}}_{i} - { }{\overline{\mathbf{x}}}_{k} } \right) $$

where ${\mathbf{x}}_{i}$ is a vector with the input variables for sample $i$; ${\overline{\mathbf{x}}}_{k}$ is the mean of class $k$; and ${\mathbf{C}}_{{{\text{pooled}}}}$ is the pooled covariance matrix between the classes. The QDA classification score for sample $i$ of class $k$ ($Q_{ik}$) is estimated using the variance–covariance for each class $k$ (${\mathbf{C}}_{k}$) in a non-Bayesian form as follows^22,25^:

4 $$ Q_{ik} = \left( {{\mathbf{x}}_{i} - { }{\overline{\mathbf{x}}}_{k} } \right)^{{\text{T}}} {\mathbf{C}}_{k}^{ - 1} \left( {{\mathbf{x}}_{i} - { }{\overline{\mathbf{x}}}_{k} } \right) $$

SVM is a powerful supervised classification method that nonlinearly transform the input sample space into a feature space using a kernel function that maximizes the margins of separation between the sample groups, and then it constructs a linear hyperplane that discriminates the samples from different groups in this feature space^23^. In this study, a radial basis function (RBF) kernel was utilized. The RBF is calculated as follows^26^:

5 $$ k\left( {{\varvec{x}}_{i} ,{\varvec{z}}_{j} } \right) = \exp \left( { - \gamma \left| {\left| {{\varvec{x}}_{i} - {\varvec{z}}_{j}^{2} } \right|} \right|} \right) $$

where ${\varvec{x}}_{i}$ and ${\varvec{z}}_{j}$ are sample measurements vectors, and $\gamma$ is a tuning parameter that controls the RBF width. In the RBF kernel function, the $\gamma$ parameter was set to 1. The SVM classification rule is obtained by the following equation^26^:

6 $$ f\left( x \right) = {\text{sign}}\left( {\mathop \sum \limits_{i = 1}^{{N_{SV} }} \alpha_{i} y_{i} k\left( {{\varvec{x}}_{i} ,{\varvec{z}}_{j} } \right) + b} \right) $$

where $N_{SV}$ is the number of support vectors; $\alpha_{i}$ is the Lagrange multiplier; $y_{i}$ is the class membership (± 1); $k\left( {x_{i} ,z_{j} } \right)$ is the kernel function; and $b$ is the bias parameter. These SVM parameters were obtained and optimized via an external validation set.

### Quality performance

The statistical parameters for the evaluation of the classification models were: accuracy (AC), sensitivity (SENS), specificity (SPEC), Youden’s Index (YOU), positive predictive value (PPV), negative predictive value (NPV), F-Score and G-Score. AC is related to the percentage of correct classification achieved by the model. SENS measures the proportion of positive results that are correctly identified while SPEC measures the proportion of negative results that are correctly identified. In this study, when we have a case–control patients approach, sensitivity can be understood as the probability to find a positive result when the disease is present, while specificity can be understood as the probability to find a negative result when the disease is not present. Youden’s index (YOU) evaluates the classifier’s ability to avoid failure. The PPV measures the proportion of positives that are correctly assigned (its value varies between 0 and 1); the NPV measures the proportion of negatives that are correctly assigned (its value varies between 0 and 1); the F-score represents the weighted average of the precision and sensitivity; and the G-score accounts for the model precision and sensitivity without the influence of positive and negative class sizes^27^. These parameters are calculated based on the equations shown in Table 3. In addition, a receiver operating characteristics (ROC) curve was generated to all models. The area under curve (AUC) value was calculated to evaluate how well the model can distinguish the samples between the different classes analysed.

**Table 3:** Equations to calculate the figures of merit for model evaluation.

Parameter (%)	Equation
Accuracy (AC)	$\frac{{{\text{TP}} + {\text{TN}}}}{{{\text{TP}} + {\text{FP}} + {\text{TN}} + {\text{FN}}}} \times 100$
Sensitivity (SENS)	$\frac{{{\text{TP}}}}{{{\text{TP}} + {\text{FN}}}}{ } \times { }100$
Specificity (SPEC)	$\frac{{{\text{TN}}}}{{{\text{TN}} + {\text{FP}}}}{ } \times { }100$
Youden’s index (YOU)	${\text{SENS}} - \left( {100 - {\text{SPEC}}} \right)$
Positive predictive value (PPV)	$\left( {\frac{{{\text{TP}}}}{{{\text{TP}} + {\text{FP}}}}} \right) \times { }100$
Negative predictive value (NPV)	$\left( {\frac{{{\text{TN}}}}{{{\text{TN}} + {\text{FN}}}}} \right) \times { }100$
F-score	$\left( {\frac{{2{ } \times {\text{ SENS }} \times {\text{ SPEC}}}}{{{\text{SENS}} + {\text{SPEC}}}}} \right)$
G-score	$\sqrt {{\text{SENS }} \times {\text{ SPEC}}}$

FN stands for false negative, FP for false positive, TP for true positive, and TN for true negative.
